# From delay to action: rethinking Chagas disease diagnosis with rapid testing in Colombia

**DOI:** 10.7189/jogh.16.03025

**Published:** 2026-08-28

**Authors:** Mario Javier Olivera

**Affiliations:** Instituto Nacional de Salud, Grupo de Parasitología, Bogotá, Colombia

**Keywords:** Chagas disease, rapid diagnostic tests, diagnosis, health equity, Colombia, neglected tropical diseases

## Abstract

Chagas disease remains one of the most underdiagnosed neglected tropical diseases in Latin America despite the availability of accurate diagnostic tools. In Colombia, the number of diagnostic tests reported between 2017 and 2023 corresponded to fewer than 4% of the estimated population at risk, reflecting persistent diagnostic inequities associated with centralised laboratory-based algorithms that are poorly adapted to rural and underserved settings. National administrative data revealed substantial geographic disparities in testing intensity, particularly in departments with the largest populations at risk. Evidence from Colombian implementation experiences and recent economic evaluations indicates that sequential rapid diagnostic test (RDT)-based strategies can substantially reduce diagnostic costs while maintaining high diagnostic accuracy and operational feasibility. Expanding access to timely diagnosis may also contribute to preventing congenital transmission and reducing the long-term burden of chronic Chagas cardiomyopathy. Integrating RDTs into a phased national diagnostic strategy represents a feasible, evidence-based opportunity to reduce diagnostic inequities and strengthen Chagas disease control in Colombia.

## DIAGNOSTIC GAP IN CHAGAS DISEASE IN COLOMBIA: COVERAGE AND INEQUITIES

Chagas disease affects an estimated six million people across Latin America [1,2]. In Colombia, national prevalence estimates range from 0.84% to 2.0% in the general population, with seroprevalence reaching up to 3.0% in high-risk subgroups, including adults and pregnant women [3,4]. Despite notable progress in vector control, chronic *Trypanosoma cruzi* (*T. cruzi*) infection remains substantially undiagnosed, particularly in rural, impoverished, and Indigenous communities where structural barriers to diagnosis persist [5].

One of the primary drivers of underdiagnosis is the silent and prolonged nature of the chronic phase, during which individuals may remain asymptomatic for years or decades [6–9]. In the absence of symptoms and systematic screening programmes, opportunities for early detection are often missed. This clinical invisibility, compounded by limited public awareness and gaps in provider training, reinforces the disease's status as a neglected tropical disease in both public perception and health system practice [10].

Although Colombia has achieved near-universal health insurance coverage, this progress has not translated into effective or equitable access to diagnostic services for Chagas disease [11,12]. National administrative records from 2008 to 2015 show that serological testing reached only 1.2% to 1.4% of the 4.8 million individuals classified as being at risk [13], with more than 85% of tests concentrated in just three departments: Boyacá, Casanare, and Santander [14,15].

### Recent trends in diagnostic coverage (2017–2023): insights from Colombia's Individual Health Services Registry data

To provide a more recent and spatially disaggregated overview, a new analysis of data from Colombia's Individual Health Services Registry was conducted for the period from 2017 to 2023 [16], during which 288,760 diagnostic tests for *Trypanosoma cruzi* infection were reported nationwide. Based on the World Health Organization (WHO) estimate of 8.1 million people at risk [4], this corresponds to an overall diagnostic coverage of only 3.6%, representing a modest improvement over previous estimates but remaining critically low relative to the size of the population requiring access to diagnosis.

**Figure 1:**
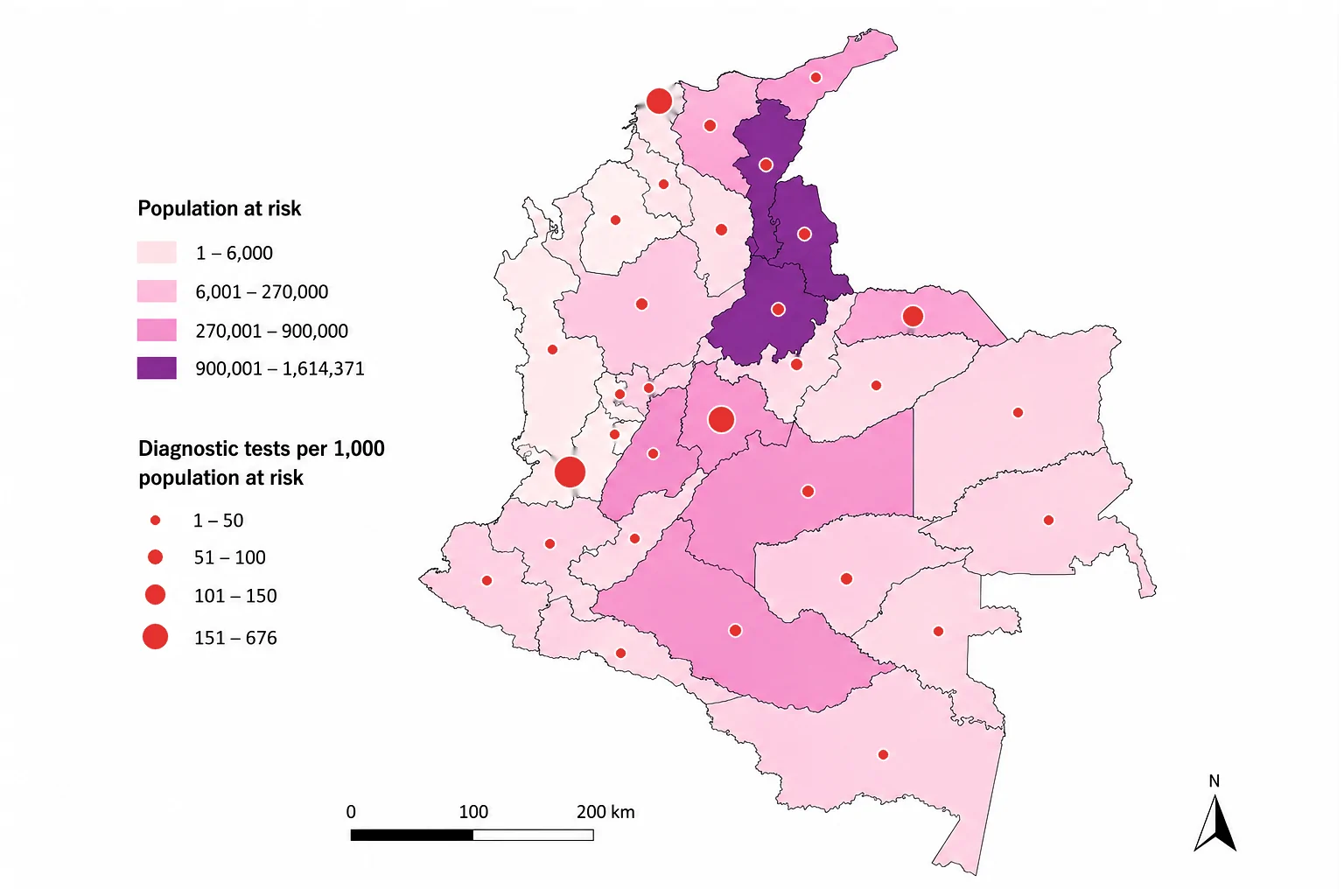
Geographic distribution of the population at risk and diagnostic testing intensity for *Trypanosoma cruzi* infection across Colombia, 2017–2023. Department shading represents the estimated population at risk, whereas circle size indicates the number of diagnostic tests performed per 1,000 population at risk using data from the Individual Health Services Registry.

Although departments with relatively small at-risk populations, such as Valle del Cauca (676 tests per 1,000 population at risk) and Atlántico (657 tests per 1,000 population at risk), reported the highest testing intensity, departments with the largest at-risk populations, including Santander (29 tests per 1,000 population at risk), Norte de Santander (14 tests per 1,000 population at risk), and Cesar (14 tests per 1,000 population at risk), showed substantially lower testing rates. These spatial disparities reveal a mismatch between diagnostic effort and epidemiological need, highlighting persistent inequities in access to diagnosis and reinforcing the urgency of implementing decentralised, context-adapted diagnostic strategies, particularly in historically underserved territories (**Figure 1**).

## STRUCTURAL BARRIERS AND REAL-WORLD LIMITATIONS OF THE CURRENT DIAGNOSTIC MODEL

The current national algorithm for diagnosing chronic *T. cruzi *infection in Colombia relies on two sequential enzyme-linked immunosorbent assays (ELISAs), with discordant results resolved through the indirect immunofluorescence assay (IFA) [17,18]. Although this protocol aligns with WHO and Pan American Health Organization (PAHO) recommendations, it is poorly adapted to the operational realities of endemic regions, where healthcare infrastructure is often limited [19].

The diagnostic process depends on stable electricity, functional cold-chain logistics, specialised laboratory equipment, and adequately trained personnel, resources that are frequently lacking in rural and remote areas [20,21]. As a result, diagnostic services remain highly centralised, with access concentrated in urban centres and national reference laboratories, excluding large segments of the population from timely diagnosis.

While ELISAs have demonstrated high analytical performance under optimal conditions, their real-world effectiveness is constrained by logistical disruptions, reagent instability, and potential cross-reactivity with *Leishmania* spp. [22–24]. A national evaluation conducted by Colombia's National Institute of Health assessed seven commercial immunoassays using 501 samples, including local *Trypanosoma cruzi* discrete typing unit I (TcI) strains. Five assays demonstrated sensitivity above 98%, and six achieved specificities above 97%. The in-house ELISA and IFA methods developed by the National Institute of Health showed 100% concordance with WHO reference panels, indicating that local *T. cruzi* genetic diversity, including TcI and TcII lineages, does not compromise performance under controlled laboratory conditions [17,25].

However, these findings should be interpreted cautiously. Under routine field conditions, critical limitations persist, including reagent degradation, supply interruptions, and the lack of point-of-care deployment. Together, these factors continue to generate structural bottlenecks. As a consequence, delays between clinical suspicion and diagnostic confirmation typically range from three to six months [18,22,23].

These operational constraints reflect a fundamental disconnect between the technical design of the diagnostic model and the environmental and logistical conditions of low-resource settings. Despite the availability of health insurance coverage and existing patient demand, the system fails to provide timely diagnosis, thereby undermining the therapeutic window for antiparasitic treatment, particularly during the early chronic phase, when clinical benefits are greatest. This misalignment contributes to persistent diagnostic inequities and reinforces the continued neglect of Chagas disease.

## RAPID DIAGNOSTIC TESTS AS A PROVEN, COMPLEMENTARY STRATEGY FOR EXPANDING ACCESS

Rapid diagnostic tests (RDTs) represent a pivotal opportunity to shift from centralised, laboratory-based diagnosis to proactive, decentralised detection of *T. cruzi *infection. PAHO endorsed the use of high-performance RDTs for Chagas disease in 2025, recommending those with ≥92% sensitivity and ≥95% specificity for screening and surveillance [26]. This endorsement marked a turning point in regional diagnostic policy, enabling countries to adopt more flexible, scalable, and operationally feasible strategies, particularly in resource-limited settings.

To assess the economic implications of integrating RDTs into Colombia's national diagnostic strategy, complementary economic evaluations were conducted under both programmatic and long-term health system perspectives. The complementary programmatic analysis conducted specifically for this viewpoint demonstrated that a sequential RDT algorithm, defined as an initial RDT followed by a second, antigenically distinct confirmatory RDT, reduced the average cost per correct diagnosis from USD 24.79 to USD 8.39, representing a 66% reduction compared with conventional enzyme-linked immunosorbent assay (ELISA)-based testing. More recently, complementary cost–utility analyses have further suggested that RDT-based diagnostic strategies may reduce lifetime costs while improving health outcomes compared with conventional diagnostic algorithms [27].

Cost estimates were based on national procurement data (2019–2023), pilot implementation reports from Boyacá and Casanare, and technical assessments by the National Institute of Health. Inputs included unit prices, training and supervision, sample transport, cold-chain requirements, and wastage rates. The analysis adopted a one-year time horizon from the health system perspective and excluded indirect costs such as productivity loss, caregiving burden, and long-term disability.

Univariate sensitivity analyses confirmed the robustness of the findings across a broad range of epidemiological and operational assumptions. Subsequent economic evaluations highlighted that the value of RDT-based strategies is driven not only by diagnostic performance but also by improvements in completion of the diagnostic cascade and reductions in loss to follow-up [27]. These findings underscore the importance of operational effectiveness when evaluating decentralised diagnostic approaches.

**Figure 2:**
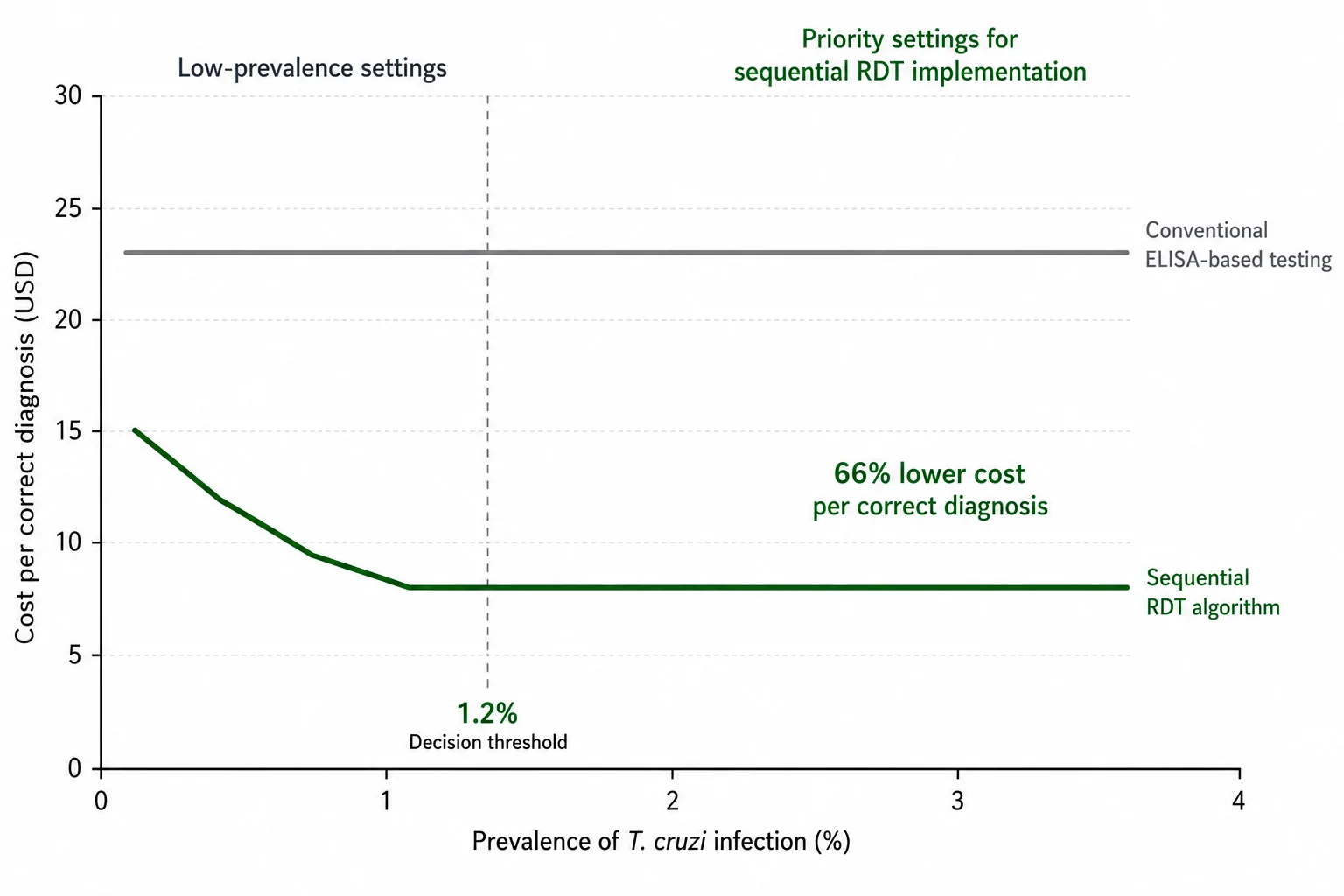
Relationship between *T. cruzi* prevalence and cost per correct diagnosis for conventional ELISA-based and sequential RDT strategies. The vertical dashed line indicates the estimated prevalence threshold of approximately 1.2% for prioritising sequential RDT implementation. Above this threshold, the economic advantage of the sequential RDT strategy increases relative to conventional ELISA-based testing. ELISA – enzyme-linked immunosorbent assay, RDT – rapid diagnostic test, *T. cruzi* – *Trypanosoma cruzi*.

RDT-based strategies demonstrated clear economic advantages in settings with a *T. cruzi *prevalence above 1.2%, reducing the cost per correct diagnosis by approximately 66% compared with conventional ELISA-based testing. The economic advantage of the sequential RDT algorithm increased progressively with higher prevalence, as the cost per correct diagnosis became increasingly favourable relative to conventional ELISA-based testing (**Figure 2**).

Beyond its economic benefits, the sequential RDT algorithm offers important operational advantages, including ease of use, rapid turnaround time, and minimal infrastructure requirements, making it particularly suitable for decentralised diagnosis in underserved and hard-to-reach settings [28,29]. A meta-analysis of 11 field-based studies reported a pooled sensitivity of 96.6% (95% confidence interval (CI) = 91.3–98.7) and specificity of 99.3% (95% CI = 98.4–99.7) for *T. cruzi *RDTs [30], comparable to conventional ELISA. Together, these findings suggest that sequential RDT-based strategies can improve both the economic efficiency and operational feasibility of Chagas disease diagnosis in endemic settings (**Table 1**).

**Table 1:** Comparison of conventional ELISA- and sequential RDT-based diagnostic strategies for chronic Chagas disease in Colombia

Domain	Conventional ELISA strategy	Sequential RDT strategy
Economic		
*Mean cost per correct diagnosis in USD*	24.79	8.39
Operational		
*Time to result*	Several days	<30 min
*Laboratory infrastructure required*	Yes	No
*Electricity required*	Yes	No
*Cold-chain dependence*	Yes	Minimal
Programmatic		
*Suitable for primary healthcare*	Limited	Yes
*Community deployment*	Limited	High
*Number of patient visits*	Multiple	Single
*Risk of loss to follow-up*	Higher	Lower

ELISA – enzyme-linked immunosorbent assay, RDT – rapid diagnostic test

**Figure 3:**
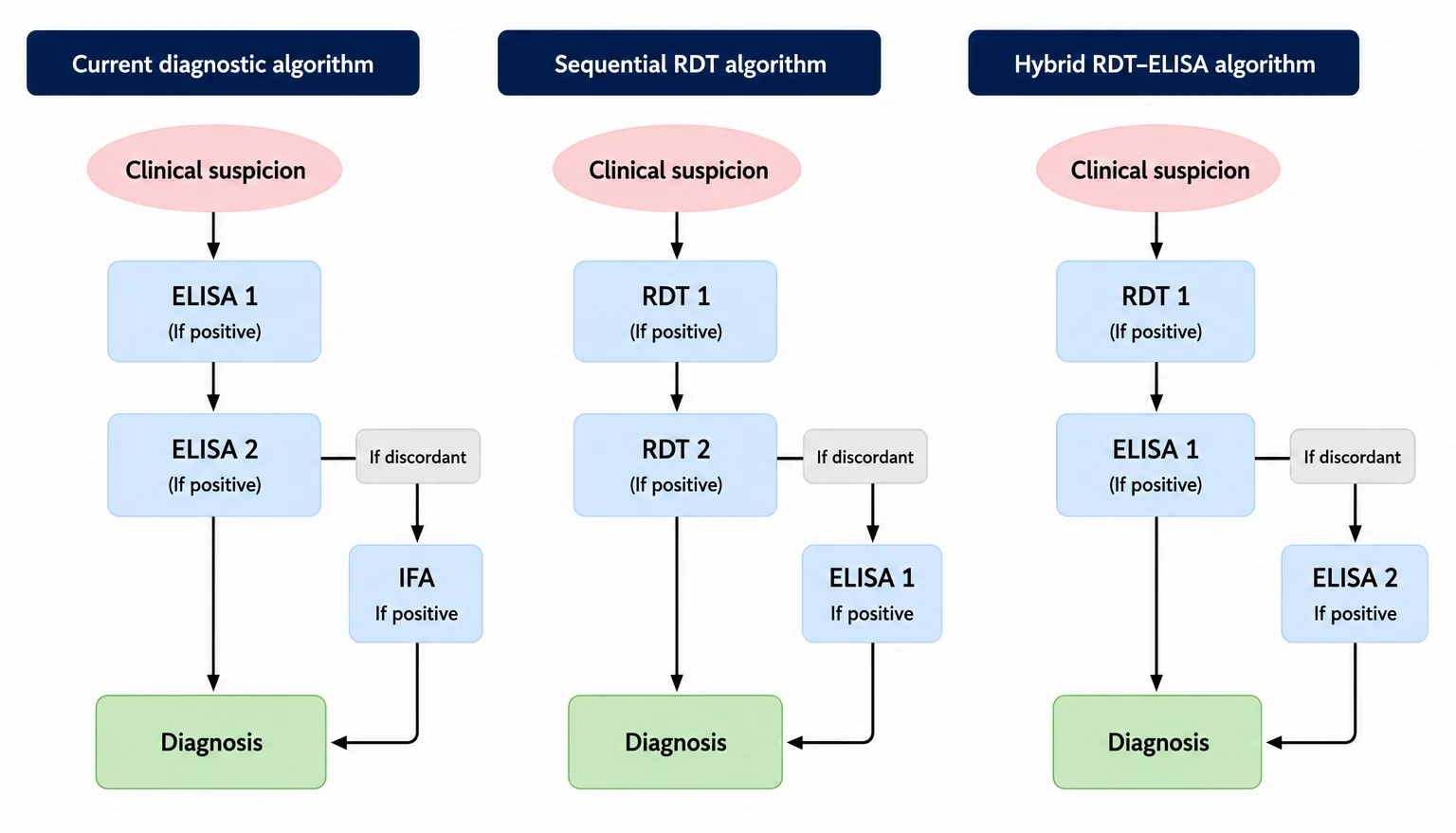
Diagnostic algorithms for chronic *Trypanosoma cruzi* infection in Colombia: conventional serology and alternative RDT-based strategies. The left panel illustrates the current diagnostic algorithm based on sequential ELISA testing with IFA for discordant results. The middle panel depicts a sequential RDT strategy, whereas the right panel shows a hybrid strategy combining RDT and ELISA before confirmatory testing in discordant cases. ELISA – enzyme-linked immunosorbent assay, IFA – immunofluorescence assay, RDT – rapid diagnostic test.

Taken together, the available evidence supports integration of RDTs as a strategic complement to, rather than a replacement for, conventional serology. Sequential RDT algorithms enable targeted expansion of diagnostic services in priority areas, improving access to timely diagnosis, increasing diagnostic coverage, and reducing geographic inequities. Earlier diagnosis of *T. cruzi* infection among women of reproductive age may additionally contribute to preventing congenital transmission, extending the benefits of decentralised diagnostic strategies beyond individual treatment to broader public health gains. Successful implementation should be guided by subnational disease prevalence, local health system capacity, and programmatic feasibility, supporting a phased and context-specific integration of RDTs into the national Chagas disease control strategy (**Figure 3**).

### Implementation enablers and operational considerations

Policy endorsement and test availability are necessary, but not sufficient. The successful deployment of RDTs requires a context-sensitive implementation framework aligned with Colombia's decentralised health system and heterogeneous epidemiological landscape [26].

Diagnostic protocols should be tailored to different levels of care, ranging from primary care posts to referral centres, and adapted to territorial risk gradients and operational capacity [24]. Health personnel should be trained in procedural aspects but also in the clinical and programmatic interpretation of weakly positive results, which remain a recurring challenge in field conditions [31].

Experiences from national malaria and HIV programmes underscore the importance of structured implementation support, including external quality assurance panels, regular supervisory visits, and ongoing refresher training. These components are essential for sustaining diagnostic performance over time and should be systematically integrated into the national RDT rollout [32].

Equally important is the integration of RDT workflows into digital health records and national surveillance platforms [33]. Automating test reporting enhances traceability, reduces transcription errors, and strengthens real-time monitoring, all of which are essential for ensuring accountability and responsiveness within the health system.

Formalising these elements within updated national guidelines would institutionalise quality assurance mechanisms, foster provider and patient confidence, and establish a stable foundation for nationwide implementation [34,35]. Ultimately, aligning technical validity with operational feasibility will be critical to ensuring that each diagnostic encounter contributes meaningfully to closing Colombia's Chagas disease detection gap in a sustainable, equitable, and measurable way.

Despite this strong technical and economic rationale, Colombia has yet to fully implement a national strategy for RDT integration. The absence of structured scale-up plans, sustained financing, and operational alignment has limited the impact of the available evidence, leaving the diagnostic gap largely unchanged. This failure to act has both clinical and economic consequences, as described below.

## THE ECONOMIC AND PUBLIC HEALTH CONSEQUENCES OF DIAGNOSTIC DELAY

Undiagnosed Chagas disease imposes a significant and preventable burden in Colombia, both human and economic. Even among the small fraction of diagnosed cases, available data reveal only a partial view of the broader, hidden impact. From a societal perspective, the estimated annual cost per person living with chronic Chagas disease is USD 4,226, encompassing direct medical expenses (USD 594), out-of-pocket costs (*e.g.* transportation, food, and laboratory tests), and substantial productivity losses due to absenteeism, presenteeism, and premature mortality [36].

The total cost attributable to diagnosed cases alone reached USD 13.1 million in 2017, representing only 1.2% of the estimated infected population and highlighting the vast scale of underdiagnosis. If all individuals living with Chagas disease were identified and treated, projected societal costs would exceed USD 140 million [36]. This extrapolated figure is not a forecast of actual expenditures but rather an estimate of the latent economic burden that remains unaccounted for in official national health planning.

The clinical consequences of diagnostic delay are also costly. Progression to chronic Chagas cardiomyopathy frequently requires advanced interventions, including pacemakers and implantable cardioverter-defibrillators, procedures that account for more than one-quarter of direct medical costs. For patients with Chagas-related heart failure, average annual healthcare expenses exceed USD 12,798 per person, largely driven by hospitalisations and cardiovascular procedures [37].

Beyond healthcare, the productivity cost of premature mortality between 2010 and 2017 was estimated at USD 29 million, with 48,621 potential years of work lost, disproportionately affecting workers in economically vulnerable sectors, including agriculture and construction [38].

In practical terms, expanding diagnostic coverage without proportional investment in treatment infrastructure may temporarily increase fiscal pressure, delaying the realisation of long-term health and economic returns. As of today, Colombia has not incorporated downstream treatment costs or surge-capacity modelling into its national Chagas disease strategy, representing a critical planning gap. Developing such projections is essential not only for sustainable financing but also for aligning diagnostic expansion with therapeutic readiness and supporting multisectoral preparedness and budgeting.

## EXTENDING THE VALUE-BASED CASE FOR RDTS

A value-based, equity-oriented approach focused on maximising health gains per resource invested reveals a substantial misalignment between diagnostic deployment and the actual geographic distribution of *T. cruzi *burden. As shown in **Figure 1**, diagnostic deployment does not align with the population at risk across departments. This mismatch highlights systemic gaps between population need and the availability of diagnostic services.

These disparities disproportionately affect historically marginalised populations, including rural, low-income, and Indigenous communities [39]. Strategic deployment of RDTs in high-prevalence, underdiagnosed areas represents not only a cost-effective public health intervention but also an opportunity to reduce long-standing diagnostic inequities. By enabling timely, community-level diagnosis, RDTs extend the reach of health services into neglected territories, thereby advancing both allocative efficiency and social justice [26].

Although this perspective did not include disease-burden-weighted cost-effectiveness modelling, future analyses should prioritise estimating the avoidable health and economic losses associated with diagnostic exclusion. These estimates would strengthen the value proposition for investment, guide geographically equitable resource allocation, and ensure that RDT scale-up reflects both operational feasibility and a sustained commitment to diagnostic equity.

## A PATH FORWARD: NATIONAL PHASED STRATEGY FOR RDT INTEGRATION

In response to the diagnostic inequities highlighted throughout this perspective, this article proposes a phased national strategy for integrating RDTs for *T. cruzi *into Colombia's health system. This approach leverages existing institutional capacity, prioritises high-burden areas and vulnerable populations, and outlines progressive milestones toward full national integration by 2030.

**Phase 1 (2025–2026): targeted introduction in high-burden municipalities**

Initial implementation should focus on endemic departments such as Casanare, Arauca, Santander, and Norte de Santander, territories where transmission persists, and diagnostic uptake remains low. RDTs would be deployed at the primary care level, with community health workers engaged in active case detection, referral for confirmatory testing, and linkage to treatment.

**Phase 2 (2027–2028): scale-up to moderate-risk settings and priority populations**

This phase would expand coverage to additional municipalities and high-risk groups, including pregnant women, older adults, and individuals with cardiovascular symptoms. Chagas screening would be integrated into maternal health and chronic disease management programmes, using both fixed and mobile teams to expand access in underserved areas.

**Phase 3 (2029–2030): consolidation and national integration**

By this stage, RDTs would be routinely available at the primary level of care, with coverage exceeding 80% in endemic municipalities. Chagas testing would be embedded in national screening protocols for antenatal care, cardiovascular risk management, and migrant health, supported by standardised diagnostic algorithms and well-established referral networks.

Key enablers for successful implementation include sustainable domestic financing, formal inclusion of RDTs in national diagnostic guidelines, continuous training and supervision, interoperable digital reporting systems, and robust subnational planning frameworks. Monitoring and evaluation mechanisms should be embedded from the outset. Core performance indicators should include RDT coverage among priority groups, positivity yield per 1,000 tests, diagnostic-to-treatment linkage rates, and user acceptability measures.

## CONCLUSIONS

Chagas disease will remain a silent and inequitable threat unless diagnostic strategies are deliberately restructured to reach populations historically excluded from routine care. RDTs are not merely technical innovations; they are strategic enablers of diagnostic equity, capable of narrowing long-standing access gaps in rural, Indigenous, and underserved areas.

Supported by robust analytical performance, demonstrated cost-effectiveness, and operational feasibility, RDTs can effectively complement Colombia's tiered diagnostic model, reducing diagnostic delays, minimising infrastructure requirements, and enabling task-shifting. This perspective does not advocate replacing conventional serology but rather integrating context-sensitive, decentralised diagnostic strategies into the national diagnostic framework, in alignment with global standards for patient-centred care.

Colombia now has the evidence, tools, and international policy consensus to implement a phased and scalable diagnostic expansion strategy. Achieving this vision will require more than political will; it will also require strong governance mechanisms, sustained domestic financing, trained personnel, interoperable digital systems, and data-driven accountability.

By integrating rapid and conventional diagnostics into a coherent national strategy, Colombia can reduce diagnostic delays, improve health system responsiveness, and prevent avoidable losses in health, economic productivity, and human potential. Moreover, Colombia's experience, grounded in data, feasibility studies, and policy readiness, can serve as a scalable model for other endemic countries in the Andean region, particularly those facing similar diagnostic inequities and decentralised health systems.

## Data Availability

**Data availability:** Data used in the 2017–2023 analysis were obtained from Colombia's Individual Health Services Registry (RIPS/SISPRO); all other data are available from the sources cited in the article.
